# The Architecture of Macular Neovascularizations Predicts Treatment Responses to Anti-VEGF Therapy in Neovascular AMD

**DOI:** 10.3390/diagnostics12112807

**Published:** 2022-11-15

**Authors:** Henrik Faatz, Kai Rothaus, Martin Ziegler, Marius Book, Georg Spital, Clemens Lange, Albrecht Lommatzsch

**Affiliations:** 1 Department of Ophthalmology, St. Franziskus Hospital, 48145 Münster, Germany; 2 Achim Wessing Institute for Diagnostic Ophthalmology, Duisburg–Essen University, 45147 Essen, Germany; 3 AugenZentrum Siegburg, MVZ ADTC Siegburg GmbH, 53721 Siegburg, Germany; 4 Department of Ophthalmology, Freiburg University Hospital, 79106 Freiburg, Germany; 5 Department of Ophthalmology, Essen University Hospital, 45147 Essen, Germany

**Keywords:** macular neovascularization, imaging, age-related macular degeneration, OCT angiography, choroidal neovascularization, anti-VEGF therapy

## Abstract

Introduction: Anti-VEGF therapy is an effective option for improving and stabilizing the vision in neovascular age-related macular degeneration (nAMD). However, the response to treatment is markedly heterogeneous. The aim of this study was therefore to analyze the vascular characteristics of type 1,2, and 3 macular neovascularizations (MNV) in order to identify biomarkers that predict treatment response, especially with regard to changes in intraretinal and subretinal fluid. Materials and Methods: Overall, 90 treatment-naive eyes with nAMD confirmed by optic coherence tomography (OCT), fluorescein angiography, and OCT angiography (OCTA) were included in this retrospective study. The MNV detected by OCTA were subjected to quantitative vascular analysis by binarization and skeletonization of the vessel using ImageJ. We determined their area, total vascular length (sumL), fractal dimension (FD), flow density, number of vascular nodes (numN), and average vascular diameter (avgW). The results were correlated with the treatment response to the initial three injections of anti-VEGF and the changes in intraretinal (IRF) and subretinal fluid (SRF) and the occurrence of pigment epithelial detachements (PED). Results: All patients found to have no subretinal or intraretinal fluid following the initial three injections of anti-VEGF showed a significantly smaller MNV area (*p* < 0.001), a lower sumL (*p* < 0.0005), and lesser FD (*p* < 0.005) before treatment than those who still exhibited signs of activity. These parameters also showed a significant influence in the separate analysis of persistent SRF (*p* < 0.005) and a persistent PED (*p* < 0.05), whereas we could not detect any influence on changes in IRF. The vascular parameters avgW, numN, and flow density showed no significant influence on SRF/IRF or PED changes. Conclusions: The size, the total vessel length, and the fractal dimension of MNV at baseline are predictors for the treatment response to anti-VEGF therapy. Therefore, particularly regarding the development of new classes of drugs, these parameters could yield new insights into treatment response.

## 1. Introduction

Age-related macular degeneration (AMD) is the most commonly cause of visual impairment in western countries [1]. It is a progressive and multifactorial disease, with the most important risk factor being aging. Genetic predisposition and lifestyle also have an influence. The late phase of the disease is characterized either by increasing loss of photoreceptor cells, retinal pigment epithelium (RPE) and choriocapillaris, termed geographic atrophy (GA) [2], or by macular neovascularizations (MNV) in neovascular AMD (nAMD), leading to exudation within or below the retina or below the RPE [3,4]. The prevalence of nAMD is 9.8% in people over 85 years of age, and based on demographic trends, projections estimate that 3.9–4.8 million people in Europe will be affected by 2040 [5]. Following the recent revision of the nomenclature, MNV were divided into three types: MNV from the choroid penetrating through Bruch’s membrane (BM) and located underneath the RPE are designated type 1; MNV growing from the choroid through the RPE into the subretinal space are referred to as type 2 MNV; and finally, MNV arising in the retina and growing in the direction of the RPE are termed type 3 MNV [6].

Local inflammation leads to drusen formation, degeneration of RPE and photoreceptors, disruption of BM, and development of MNV [7]. Cytokines, chemokines, leukocytes, macrophages, microglia, and the complement system are involved [8]. Many components are involved, such as tumour necrosis factor alpha, interferon-gamma and -beta, interleukins-4, -5, -6, -8, -10, -13 and -17, transforming growth factor beta, monocyte chemoattractant protein-1, and VEGF [9]. Cytokines can drive the formation of VEGF and thus further promote MNV development [10]. Currently, the only therapeutic option is to inhibit VEGF and, therefore, reduce or stop the progression of the disease, although a full remission is not possible.

Patients with nAMD usually require treatment over a period of years with frequent intravitreal anti-vascular endothelial growth factor (VEGF) injections and follow-up visits [11]. Patients differ widely with regard to both the necessary frequency of injections and how their vision evolves over the period of treatment [12], making prediction of the course very difficult. Fibrosis or atrophy of the macula also occurs in one third of patients, despite anti-VEGF therapy [13]. Therefore, it would be desirable for these patients to be identified at an early stage and for further therapeutic options to be available in the future to achieve a better visual outcome through more individualised therapy. Alongside ophthalmoscopy and measurement of central visual acuity, the gold standard for diagnostic imaging is fluorescein angiography (FA) with spectral-domain optical coherence tomography (SD-OCT) [14]. Indocyanine green angiography (ICGA) is very helpful in cases of unclear MNV type. However, with FA and ICGA, the dye has to be applied intravenously, which is uncomfortable for the patient, and there may be local as well as systemic side effects. In very rare but severe cases, even death can result from allergic shock.

Optical coherence tomography angiography (OCTA), a modality developed from optical coherence tomography (OCT), detects the movement of blood cells and renders this motion as a grayscale image in contrast to the adjacent static tissues. The retinal and choroidal blood vessels are thus depicted noninvasively [15]. A further advantage of OCTA over FA is that the vessels can be viewed segment-wise and in three dimensions, with the vascular structure portrayed in detail [16]. This permits objective mathematical description of MNV and shows previously unseen differences in patterns of disease [17,18,19]. It would be desirable to have a biomarker before the start of anti-VEGF-therapy that predicts the response to therapy in order to enable optimal patient education and individual therapy. Patients differ widely with regard to both the necessary frequency of injections and how their vision evolves over the period of treatment, making prediction of the course very difficult.

The goal of the study was to evaluate the hypothesis that there are characteristics of the vascular architecture of MNV at the time of diagnosis by OCTA and quantification of the MNV that yield pointers to whether the patient will have an active or inactive MNV after the initial three injections of anti-VEGF. A further analysis was conducted to determine whether the vascular parameters of MNV are correlated with changes in the distribution of subretinal fluid (SRF) and intraretinal fluid (IRF) or the occurrence of pigment epithelial detachments (PED).

## 2. Materials and Methods

The study was conducted in adherence to the tenets of the Declaration of Helsinki and was approved by the ethics committee of the Westphalia—Lippe Medical Association and the University of Münster. All patients who took part gave their written consent.

Study Population This was a retrospective analysis of consecutive patients with newly diagnosed nAMD. General information such as age, sex, best corrected ETDRS-visual acuity, and history of other ocular diseases was recorded for all patients. In all participants, nAMD was diagnosed by means of FA and SD-OCT (Spectralis© HRA + OCT, Heidelberg Engineering, Heidelberg, Germany) and clinical examination. Each diagnosis was verified by a senior grader at the Reading Center of M³ Macula Monitor Münster on the basis of the characteristic appearance of the various types of MNV on multimodal imaging [6]. A clinical examination with ophthalmoscopy and SD-OCT imaging was also performed at follow-up after upload with anti-VEGF. These were used to make the decision regarding current disease activity. Furthermore, the presence of IRF and SRF was described, as well as the presence of PED. Patients were treated with ranibizumab, aflibercept, or bevacizumab, which showed comparable results in their efficacy in nAMD patients [20,21,22].

We investigated 169 eyes of 158 treatment-naïve patients with MNV in nAMD. Sixty-one of these eyes were excluded because the signal strength was insufficient for precise quantification, the MNV was not completely visualized, or the vascular structure of the MNV was obscured or distorted by artifacts. Of the remaining 108 eyes in further 18 eyes, no MNV could be distinguished despite good image quality. Ninety eyes remained for analysis.

OCTA Image Analysis In addition to OCT and FA, all patients underwent OCTA using the swept-source OCTA device PLEX^®^ Elite 9000 (Carl Zeiss Meditec, Dublin, CA, USA) at the time of diagnosis. At a wavelength of 1060 nm and a rate of 100 000 A-scans/s in a 6×6-mm field, this apparatus obtains two consecutive B-scans each with 500 A-scans. Automatic suppression of artifacts was selected, because this setting improves the visualization of MNV and thus their quantification [23]. For analysis of the MNV, we selected segmentation from the outer retina to the choriocapillaris (ORCC: 0 µm from the outer plexiform layer to 49 µm below BM). Because morphological changes in the retina often led to incorrect segmentation, all B-scans showing the MNV were double-checked and the segmentation lines corrected manually as and when required.

**Figure 1:**
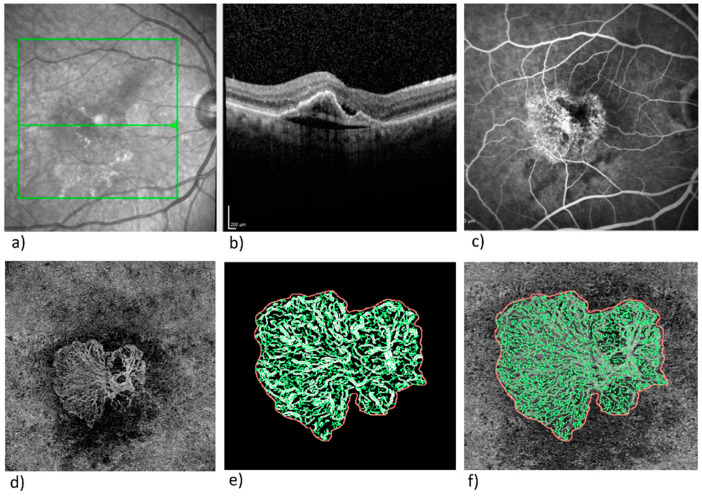
Typical images from patients with newly diagnosed nAMD: (**a**) IR-image with OCT line; (**b**) SD-OCT image; (**c**) FA; (**d**) en-face OCTA in ORCC segmentation; (**e**) binarized MNV; and (**f**) skeletonized MNV.

In the exported en-face OCTA images, the MNV was delineated in the en-face view using the program Fiji (National Institute of Mental Health, Bethesda, MD, USA), and the MNV were isolated from the rest of the image and saved for further analysis. The vascular network of the MNV was extracted via MatLab (Mathworks, Version R2014b; Natick, Massachusetts, USA). Skeletonization of the vascular network was achieved by multiscale calculation of the gradient field in the en-face OCTA image. This technique, described in detail elsewhere [24,25], depicted both wide and very narrow vascular segments as unbroken midlines. Next, the diameter of every vascular segment was measured. The following six vascular parameters were chosen for morphological characterization of the MNV structure: area, flow density, fractal dimension (FD), total vascular length (sumL), number of vascular nodes (numN), and average vascular diameter (avgW). The parameters numN, avgW, and FD describe the MNV architecture directly and provide information on the degree of branching in the MNV and its maturity. FD is a mathematical measure of the overall complexity of a structure and has previously been identified as a significant parameter of changes during anti-VEGF therapy [26,27]. Flow density is a measure of the proportion to which an MNV is vascularized and can be shown by all commercially available OCTA devices; it can thus be analyzed with no need for complex image processing. Figure 1 illustrates multimodal imaging (FA, SD-OCT, and OCTA) in the right eye of a patient with newly diagnosed nAMD together with the images resulting from binarization and skeletonization of the data.

Assessment of disease activity and analysis of SRF, IRF, and PED ensued after the upload phase of SD-OCT.

Inclusion Criteria Only patients with an initial diagnosis of nAMD with type 1, 2, or 3 MNV were included. The visual acuity had to be at least 1.3 logMAR and the patient had to agree to anti-VEGF therapy after a detailed explanation. The upload period with 3-times intravitreal anti-VEGF administration had to take place at four-week intervals. The patient had to understand the conditions of study participation and give written consent.

Exclusion Criteria Patients with inadequate OCTA image quality (quality score < 6) were excluded, as were those with any retinal pathology other than nAMD. We also excluded patients with additional retinal diseases, e.g., diabetic retinopathy or retinal vascular occlusions, and those in whom OCTA detected no MNV or was disrupted by artifacts. Moreover, patients were excluded with aneurysmal type 1 MNV (polypoidal choroidal vasculopathy).

Statistics The data were evaluated with the statistics software R (version 4.0.2). The level of significance was set at 5% for all analyses. First, we tested the homogeneity of variance (Levene test) and the normal distribution of the residuals (Shapiro—Wilk test). To describe the location and spreading parameter of the variables, we exposed the mean and standard deviation (in case of normally distributed data), and the median with the 25% and 75% quartile (non-normal distribution). The differences between the two groups were tested for significance using the Wilcoxon rank-sum test. This was followed by adjustment of the *p*-values according to Benjamini–Hochberg.

## 3. Results

Ninety eyes remained for analysis. As classified on SD-CT, FA, and OCTA, 39 MNV were type 1, 32 were type 2, and 19 were type 3. The patients’ mean age was 78.1 ± 6.9 years (55 female and 35 male), and their best-corrected visual acuity was 0.59 ± 0.33 logMAR at initial diagnosis and after the upload treatment with anti-VEGF visual acuity was significant improved 0.49 ± 0.33 logMAR (*p* < 0.0001).

**Figure 2:**
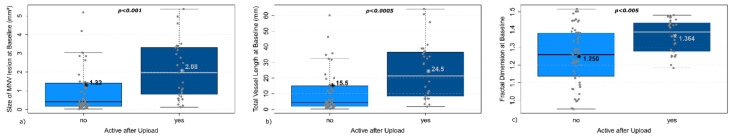
Boxplots showing the distribution of the area (**a**), total vascular length (**b**), and fractal dimension (**c**) of the MNV at the time of initial diagnosis in relation to the activity status after three intravitreal injections of anti-VEGF. Each dot represents an individual measurement. The boxes enclose the measurements between the uppermost and lowermost quartiles. The whiskers show the last measurements within the 1.5-fold interquartile range. * = Mean, bar = median; Wilcoxon rank-sum test.

Three of the MNV vascular parameters evaluated at the time of diagnosis differed significantly between the patients with inactive and those with active MNV following the upload phase: The eyes with no activity after treatment showed (1) a significantly smaller MNV area (median 0.42 mm², confidence interval [0.18–1.38]) than those eyes with persistent activity (median 1.96 mm² [0.81–3.31]), which frequently still displayed exudation (*p* < 0.001, Figure 2a); (2), a significantly lower sumL (median 4.39 mm [1.93–14.97] versus 21.39 mm [8.43–36.58]; *p* < 0.0005, Figure 2b); and (3) a significantly lower FD (median 1.25 [1.14–1.38] versus 1.39 [1.28–1.44]; *p* < 0.005, Figure 2c).

The remaining vascular parameters showed no significant differences between the eyes with a dry retina following the upload phase and the eyes with recalcitrant exudation: median avgW 21.89 µm [19.87–24.45] in patients without activity, 21.88 µm [20.37–23.54] in those with persistent activity (*p* = 0.7); median numN 470.20 [441.04–502.10] versus 464.71 20 [434.96–494.58] (*p* = 0.67); and median flow density 40.52 [38.03–42.36] versus 40.92 [39.35–43.14] (*p* = 0.56).

The MNV vascular parameters with a significant effect on the activity status after upload were analyzed following the adjustment of the *p*-values according to Benjamini–Hochberg with regard to the changes in SRF, IRF, and PED.

**Figure 3:**
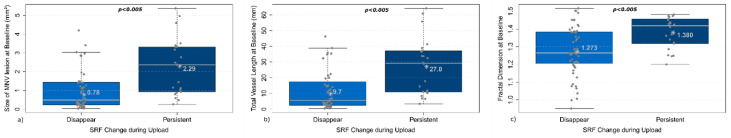
Boxplots showing the distribution of the area (**a**), total vascular length (**b**), and fractal dimension (**c**) of the MNV at the time of initial diagnosis in relation to the absorption or persistence of subretinal fluid after three intravitreal injections of anti-VEGF. Each dot represents an individual measurement. The boxes enclose the measurements between the uppermost and lowermost quartiles. The whiskers show the last measurements within the 1.5-fold interquartile range. * = Mean, bar = median; Wilcoxon rank-sum test.

The MNV of patients with persistent SRF following the intravitreal anti-VEGF upload phase had a significantly larger area (*p* < 0.005, Figure 3a), greater sumL (*p* < 0.005, Figure 3b), and higher FD (*p* < 0.005, Figure 3c) before treatment than those in patients with fully resorbed SRF. Area, sumL, and FD before treatment had no significant effect on the resorption/persistence of IRF after upload.

**Figure 4:**
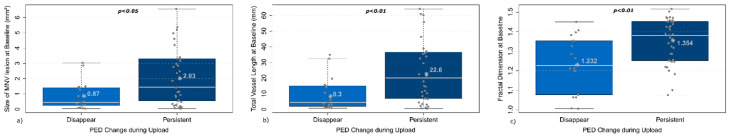
Boxplots showing the distribution of the area (**a**), total vascular length (**b**), and fractal dimension (**c**) of the MNV at the time of initial diagnosis in relation to the resolution or persistence of pigment epithelial detachment after three intravitreal injections of anti-VEGF. Each dot represents an individual measurement. The boxes enclose the measurements between the uppermost and lowermost quartiles. The whiskers show the last measurements within the 1.5-fold interquartile range. * = Mean, bar = median; Wilcoxon rank-sum test.

The MNV of patients with persistent PED after the upload phase showed a significantly larger area (*p* < 0.05, Figure 4a), greater sumL (*p* < 0.01, Figure 4b), and higher FD (*p* < 0.01, Figure 4c) than those of patients with completely resolved PED.

## 4. Discussion

The disease course and the response to anti-VEGF therapy vary considerably among patients with nAMD. Numerous studies have studied predictive biomarkers for the need of treatment or the evolution of visual acuity [28,29,30,31]. Although most of these studies used SD-OCT to identify biomarkers [28] and thus study rather secondary effects such as SRF or IRF, the present study focusses on the MNV architecture using OCTA at baseline to determine potential predictors for treatment response.

The efficacy of anti-VEGF therapy with ranibizumab, aflibercept, and bevacizumab can be considered equivalent and shows significant visual acuity improvement, as we also found in our study population [20,21,22,32,33]. The challenge in the further course of the disease is to maintain the initially achieved visual improvement. This requires many control examinations and treatments. The challenge is to treat the patients individually exactly when it is necessary. Both over- and under-treatment should be avoided. Since the treatment criteria SRF, IRF, and PED are only secondary effects of MNV, the MNV vascular architecture could provide direct information about the stage of the disease.

We found a significant impact of MNV area and sumL on the response to anti-VEGF therapy in our cohort. Both of these two parameters delineate MNV size: the area describes the horizontal extent of an MNV below and within the retina, whereas sumL is the total cumulative length of the vessels in the MNV. The fact that larger MNV with higher sumL values still often show signs of activity in the form of persistent fluid after three injections of anti-VEGF agrees with our assumption that these MNV have been evolving over a longer period of time and thus exhibit greater disease activity or are more resistant to treatment. Previous studies have also shown that the occurrence of SRF at initial diagnosis is associated with a larger area and longer total vessel length of MNV [19,34]. It was thus to be expected that MNV, which still present fluid after the upload phase, would have a higher avgW. In our study population, however, this was not the case. One reason could be the technical limitation of image resolution. In 6×6 mm scans a pixel represents 20 µm, hence, more precise differentiation is impeded. Moreover, in terms of avgW, the presence of longstanding, prominent vessels may be balanced by large numbers of capillaries, as can be assumed in highly active lesions. Another reason could be that the more prominent MNV vessels form only after a protracted period of time and may still develop during extended anti-VEGF therapy, as observed by Sulzbacher et al. [35].

Our study also revealed that the FD was significantly greater in MNV that continued to show signs of activity after the upload phase. As a higher FD represents in favor of a more complex vascular structure, this could be explained by higher activity despite anti-VEGF therapy. Al-Sheikh et al. also showed that FD can be used as a parameter for assessing the activity of MNV, and that a higher FD is associated with activity. Furthermore, they concluded that FD changes differently in the inner and outer regions of MNV after anti-VEGF therapy [26].

In contrast, no significant differences were found with respect to flow density or numN. The flow density characterizes the percentage of the MNV area that is vascularized. Commercially available OCTA devices can calculate this proportion automatically for a defined area, so that no complex image processing is required to obtain the flow density. Nevertheless, flow density is not appropriate for description of the vascular architecture, because the presence of a large number of small vessels can yield the same value as one single large vessel. Similarly, although numN states the number of vascular nodes, it may not be helpful in the presence of different compositions of the MNV. This parameter might be more useful if, for example, the central and peripheral portions of MNV were analyzed separately, or if three-dimensional representation were possible.

Our analysis of the resolution or persistence of SRF and IRF and the presence or absence of PED following anti-VEGF therapy showed a significant effect of greater MNV area, sumL, and FD on the persistence of SRF and presence of PED. Older MNV thus give rise to more treatment-resistant morphological alterations of the retina. MNV with treatment-resistant SRF seem to exert a protective effect on the growth of geographic atrophy and are associated with better vision, so under certain circumstances, SRF is tolerated [22,36]. PED, however, are associated with poorer vision in the long term, especially if the RPE tears in the course of the disease [37]. The presence of IRF also goes along with a negative effect on visual outcome [22,28]. However, none of the vascular parameters investigated in our study cohort had a significant influence on the persistence or complete resolution of IRF. Lee et al. have shown that by means of deep learning, an artificial network is able to predict the treatment response of patients with nAMD on the basis of scans from SD-OCT, FA, and indocyanine green angiography [38]. In a post-hoc analysis, Schmidt-Erfurth et al. sought to identify biomarkers in the imaging material of large data sets from clinical studies. The described characteristics of the OCT images predominantly represented secondary effects of MNV, such as SRF/IRF, RPE alterations, PED, atrophy, hyperreflective foci, subretinal hyperreflective material, and changes in retinal thickness [28]. OCTA provides new detailed information on the vascular architecture of MNV, enabling more precise delineation of the various MNV types [17,18]. Schranz et al. also described the vascular architecture of MNV in detail in a study and investigated at initial diagnosis and after one month (after single anti-VEGF administration) whether the MNV morphology or its change due to therapy allows conclusions to be made about the fluid distribution [34]. However, no correlations could be found in the study population, which could possibly be due to the too short examination interval of one month. Thus, in the pivotal studies, an initial administration of three months is recommended in order to achieve the greatest treatment success [39]. The further processing of the OCTA data also depends on the devices and the software used for vessel analysis, which can lead to differences in the results. Furthermore, one study rated the OCTA information for a machine-learning algorithm as more important than the OCT information for the decision regarding the active or inactive status of MNV [40].

Future studies should also incorporate OCTA imaging data, because multimodal imaging improves prediction accuracy and thus facilitates more individualized treatment and follow-up. Furthermore, it would also be interesting to obtain data on systemic changes, such as inflammation levels, in addition to the individual MNV vessel configuration, in order to better classify patients and possibly provide them an individualised therapy [9].

Our study has some limitations. For example, we analyzed only 6 × 6 mm OCTA scans in all patients, because smaller images often fail to show the entire MNV. However, the larger the scan, the lower the image resolution. As we found previously, 3 × 3 images depict the vascular distribution in more detail [41]. Moreover, OCTA detects the blood flow only for a particular time window; the flow before and afterwards is not taken into account. Future advances in technical performance will undoubtedly improve image quality. Another limitation is that the OCTA technique is impeded by the weakening of the signal in the presence of opacities of the cornea, lens, vitreous body, or retina. It would also be desirable to develop the capacity to view MNV in three dimensions, enabling more precise depiction and analysis of their structure. Thus, it is unclear to which extent the vessels imaged by OCTA correspond to the real vessel structure. Further development of the technology will probably improve the imaging in the future in order to get closer to reality.

The goal for future research is to confirm our finding in larger cohorts of patients and to take advantage of machine learning to transform OCTA-derived data into knowledge. The algorithms can conduct automatic searches of large data sets to identify characteristics that reliably predict the outcome. In this way, machine learning will improve our diagnostic capabilities and support treating physicians in clinical decision making with respect to the diagnosis and treatment of their patients [42].

## 5. Conclusions

In summary, OCTA yields data on MNV that improve the prediction of treatment response. The most important parameters in this regard are MNV size and total vascular length, but the complexity of MNV-represented by the fractal dimension also plays an important role. In future studies, these data derived from OCTA could contribute to the reassessment of existing and novel medications and enable the individualized treatment of nAMD through prediction of the outcome.

## Data Availability

All data used to support the findings of this study are included within the article and are available from the corresponding author upon reasonable request.
